# Musculoskeletal ultrasound-guided needle knife therapy in the treatment of refractory nonspecific low back pain: A single-blind, randomized controlled trial

**DOI:** 10.1097/MD.0000000000041066

**Published:** 2024-12-27

**Authors:** Xia Li, Hongkai Zhang, Sidi Zhang, Mingqi Wu, Shiyun Wang, Zhanying Tang, Jing Xiao

**Affiliations:** a Fenglin Community Health Service Centre, Shanghai, China; b Longhua Hospital, Shanghai University of Traditional Chinese Medicine, Shanghai, China; c Jinshan District Hospital of Integrated Chinese and Western Medicine, Shanghai, China.

**Keywords:** JOA score, multifidus muscle, musculoskeletal-guided ultrasound, needle knife therapy, NRS score, ODI score, refractory nonspecific low back pain

## Abstract

**Background:**

This clinical study aimed to evaluate the Numerical Rating Scale (NRS), Oswestry disability index (ODI), and Japanese Orthopedic Association (JOA) scores at 2, 4, 6, and 12 weeks, and the thickness of the 4th lumbar multifidus under ultrasound at 6 and 12 weeks, using musculoskeletal ultrasound (MU)-guided needle-knife loosening therapy for treating refractory nonspecific low back pain (RNSLBP) compared to usual care, to demonstrate the clinical efficacy of needle knife therapy for RNSLBP. This study used a single-blind, randomized controlled design.

**Methods:**

A total of 66 patients with RNSLBP who met the inclusion criteria were randomly divided into an observation group and a control group of 33 patients. The observation group underwent MU-guided needle knife release for RNSLBP, whereas the control group underwent low-frequency transcutaneous electrical nerve stimulation therapy, exercise therapy, and oral celecoxib capsules if necessary.

**Results:**

No statistically significant differences were observed in the baseline data (sex, age, body mass index, disease duration, NRS score, ODI score, JOA score, and 4th lumbar multifidus muscle thickness) (*P* > .05) between the 2 groups, with both groups having flat baseline and comparable indices. After 2, 4, 6, and 12 weeks of treatment, NRS and ODI scores decreased, while JOA scores increased in both groups, with statistically significant differences both within and between the 2 groups. The observation group showed better results than did the control group. The difference in multifidus muscle thickness between the 2 groups was not statistically significant after the final treatment; however, at the 12-week follow-up, the observation group showed significantly greater multifidus muscle thickness than the control group, with a statistically significant difference.

**Conclusion:**

This study demonstrated that MU-guided needle knife release effectively treats RNSLBP by reducing pain, improving lumbar spine function, and increasing the multifidus muscle thickness. It is efficient, safe, has a shorter treatment period, and causes fewer adverse reactions.

## 1. Introduction

Low back pain (LBP), referring to acute and chronic pain in the posterior region of the lumbar gluteal area from the 12th rib margin down to the subgluteal crease, is a common clinical condition that is classified into 2 major categories: idiosyncratic (caused by a specific etiology of spinal or non-spinal origin) and nonspecific.^[1]^ Determining the cause of LBP symptoms is difficult for most patients with chronic low back pain (CLBP); hence, they are usually categorized as chronic nonspecific low back pain (CNSLBP).^[2,3]^ CNSLBP is characterized by nociceptive pain associated with mechanical stress or injury to the soft tissues (muscles, ligaments, and fascia) during low back activity. This persistent and recurrent CNSLBP is usually caused by the disruption of the motor control system of the stabilizing muscles of the lumbar spine and an imbalance in their function, followed by vertebral joint overload.^[4]^ Surgery was not indicated for this group of patients. Instead, treatment aims to relieve LBP through noninvasive therapies (physical therapy, exercise and massage, etc.) or pharmacological treatments (such as nonsteroidal anti-inflammatory and analgesic drugs).^[2]^ However, these pain management techniques do not achieve satisfactory results in this patient population, and the quality of evidence for their effectiveness is low.^[5–7]^ Moreover, patients with CNSLBP who do not respond to traditional pain management techniques are categorized as having refractory nonspecific low back pain (RNSLBP).^[8]^ According to the literature, RNSLBP can be diagnosed in patients with CLBP who have had LBP symptoms for at least half of the past year and have undergone more than 90 days of medical intervention (pharmacological intervention plus at least 1 type of physical therapy) without relief.^[9]^

**Figure 1.**
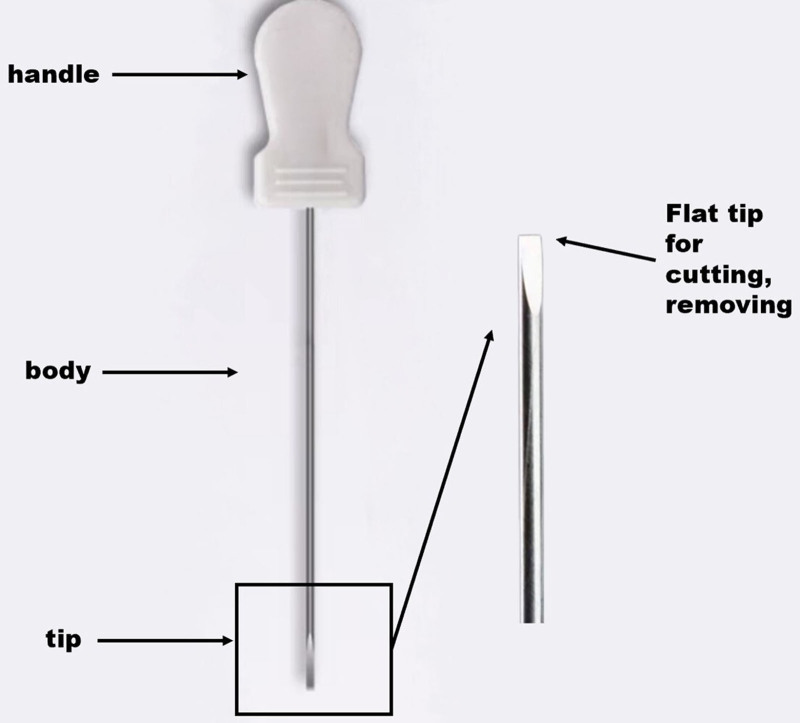
Needle knife schematic.

According to meta-analysis results, acupuncture therapy is clinically significant for treating CNSLBP.^[10]^ Needle-knife therapy, a form of acupuncture therapy, combines the holistic concepts of Chinese medicine with precise methods of Western medicine. The needle knife has the advantages of minimal invasiveness, strong targeting, high safety, few adverse reactions, and short recovery time in the treatment process (Fig. 1).^[11]^ Published trial results^[9]^ indicate that RNSLBP can be treated by stimulating the multifidus muscle to cause episodic contraction and that needle-loosening treatment can relieve muscle spasms, and improve local tissue microcirculation through the needle body directly stimulates the multifidus muscle, thus improving the clinical symptoms of patients with RNSLBP.^[12]^ Patients with CNLBP are often accompanied by insufficient low back core strength, poor stability, and morphological changes in the lumbar paraspinal muscles, such as decreased muscle thickness, decreased cross-sectional area and increased fat infiltration, among which the changes in the multifidus and erector spinae are more obvious.^[13,14]^

Musculoskeletal ultrasound (MU) uses a special high-frequency ultrasound probe to assess and diagnose conditions affecting muscles, tendons, fascia, other soft tissues, and skeletal lesions.^[15]^ MU-guided needle-knife loosening treatment allows dynamic and clear visualization of the needle and the lesion, enabling precise targeting of the lesion site. Real-time monitoring ensures proper loosening of the lesion tissue to avoid excessive treatment and prevent damage to nerves, blood vessels, and other tissues, thereby reducing the risk of intraoperative adverse reactions associated with blind needling.^[15–18]^

This study aimed to precisely stimulate the damaged multifidus muscle through the MU-guided needle-knife to induce muscle contraction, thereby treat RNSLBP. This clinical trial will evaluate the clinical efficacy and safety of MU-guided needle-knife loosening for RNSLBP in comparison with usual care through a randomized and controlled trial.

## 2. Materials and methods

This study used a single-blind, randomized controlled design. The study participants were patients who met the inclusion criteria for RNSLBP, signed an informed consent form, and were willing to undergo treatment. The study was reviewed and approved by the Ethics Committee of Longhua Hospital, affiliated with Shanghai University of Traditional Chinese Medicine, with the ethical number [2023-LHXS-077], and registered with the International Traditional Medicine Clinical Trial Registry (http://itmctr.ccebtcm.org.cn/; registration number: ITMCTR2024000200). Recruitment began on March 1, 2023, and ended on October 30, 2023.

According to the China Clinical Guidelines for Nonspecific Low Back Pain^[19]^ and related literature,^[9]^ the inclusion criteria were as follows: cooperation with treatment without cognitive dysfunction; a diagnosis of CNSLBP; LBP duration ≥ 12 months, with recurrent episodes or persistent pain for ≥ 6 months; ≥90 days of medical interventions; Numerical Rating Scale (NRS) scores of 4 to 8 (0–10); Oswestry Disability Index (ODI) scores of 13 to 36 (range: 0–50); positive lumbar segmental stability test in the prone position; and no treatment related to RNSLBP for 2 weeks before the study. Patients were excluded if they had specific LBP disorders: spinal, neurologic, visceral system, vascular, and cardiac disorders; comorbid psychiatric, language, and/or cognitive disorders preventing voluntary cooperation with treatment and follow-up visits; a history of previous low back surgeries; pregnancy or lactation; and injury at the site of application and serious infection.

### 2.1. Randomization and blinding

#### 2.1.1. Random sequence generation and grouping methods

Participants in this clinical trial were randomly assigned using the random number table method. Random numbers were generated using SPSS Statistics (version 25.0; IBM, Chicago) to create a random grouping sequence that allocated participants in a 1:1 ratio to either the observation group receiving needle-knife relaxation treatment or the control group receiving usual care.

#### 2.1.2. Blind setting

Owing to the specific nature of this pilot study, double blinding of the administrators and participants was not feasible; hence, this trial was single-blinded. Once the randomized groups were formed, the evaluators performed the appropriate assessments without knowledge of the group assignments. Statistical data analysts collected, summarized, and analyzed the data from the case report form using SPSS Statistics software (version 25.0) to ensure objective and accurate data analysis.

### 2.2. Procedures

**Figure 2.**
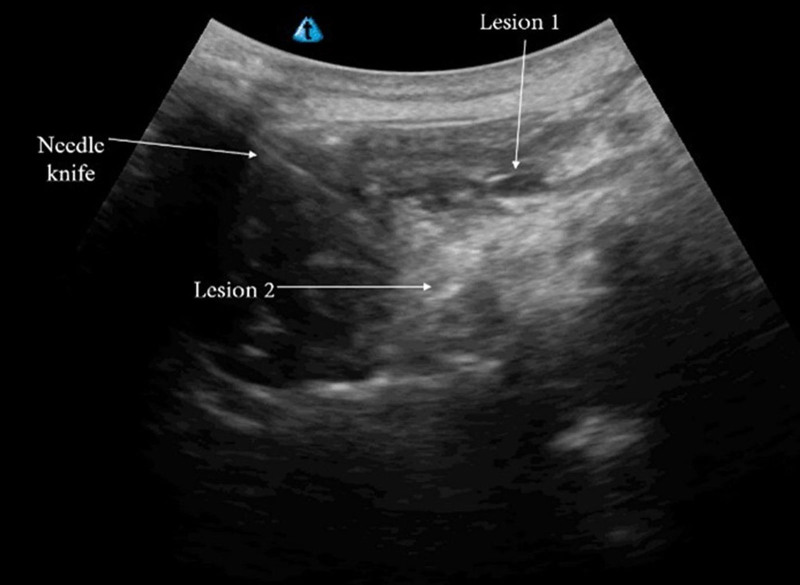
Multifidus muscle visualization under MU. MU = musculoskeletal ultrasound.

The observation group was treated with MU-guided needle-knife release of the multifidus muscle (Fig. 2).

1. Confirmation of the point: The patient lay prone on a treatment bed, fully exposing the lumbosacral region. A coupling agent was applied using a uSmart3300 portable color ultrasound system high-frequency probe. The probe was initially placed parallel to the spinal long axis perpendicular to the skin so that the central part of the probe was on top of the spinous process and then moved forward from the sacral vertebrae to the short-arc-shaped longitudinal structure connected with the peak-like spinous process. The probe was continuously moved forward until the fourth lumbar vertebra appeared in the image field of view. Subsequently, the probe was moved transversely perpendicular to the long axis of the spine to obtain an ultrasound image of the multifidus muscle and to identify and mark the focal points of the knotted tendon.
2. Preoperative preparation: The standardized disinfection procedure was conducted around the marked points. A sterile cavity towel was placed, the operator donned sterile gloves, the probe was covered with a sterile protective casing, sterile coupling agents were applied, and local infiltration anesthesia was administered using 10 mL of 1% lidocaine.
3. MU-guided needle-knife release: The assistant held the MU probe to cover the labeled points, fully revealing the lesion in the display. The operator held a sterile, single-use 0.8 × 50 mm needle knife, with the cutting edge parallel to the long axis of the spine, and quickly inserted the needle into the skin at the front of the probe. Observed in real time on the MU display, the needle knife releases diseased muscle tissues. Meanwhile, the patient usually feels soreness and pain at the needle insertion site, and the operator can hear the cutting sound and feel resistance of the muscles through the needle knife. Muscle twitching can be observed if the excruciating pain point is loosened. The operator proceeded to loosen, clear, and dredge the adhered lesions. Once MU observation confirmed complete loosening of the lesion, the needle knife moved smoothly without blockage, and the muscle at the excitation point was no longer twitched; the needle knife was withdrawn.
4. Post-operative treatment: After withdrawing the needle, the needle hole was pressed for 1 to 2 minutes to stop bleeding, and the wound was covered with a sterile dressing. The patient was asked to keep the wound clean and dry for 24 hours.

Needle-knife treatment was carried out once every 2 weeks, and 3 consecutive courses of treatment were carried out for a total of 6 weeks.

The control group was treated with usual care,^[20–22]^ consisting of low-frequency transcutaneous electrical nerve stimulation combined with lumbar spine exercise training methods and oral celecoxib capsules for pain relief, if necessary.^[23]^

Low-frequency transcutaneous electrical nerve stimulation^[22]^: The patient was placed in a prone position, exposing the treatment area. Electrode sheets were placed at the center of the pain in the lumbar back, and conductive electrode sheets were placed on both sides, with the intensity set to the patient’s maximum tolerance. Each session lasted 15 minutes, and the output current was adjusted based on the patient’s tolerance. Treatments were administered 3 times a week every 2 weeks, with each course lasting 2 weeks for 3 courses over 6 weeks.

The exercise therapy^[24]^ process was as follows:

1. The patient was positioned supine, holding one lower limb as close to the sternum as possible while flexing to the maximum range, and keeping the other lower limb straight for 5 seconds . This was alternated between left and right and repeated 10 times.
2. The patient was in a supine position, held both knees as close to the sternum as possible, flexed to the maximum range, and holding for 5 seconds before returning to the lying position, again holding the knees. This procedure was repeated 10 times.
3. In the supine position with limbs, the patient naturally extended and relaxed, flexing the head and neck upward until the scapula was lifted off the bed surface. They held this position for 5 seconds before lying down. This upward motion was repeated 10 times.
4. The patient lay in the supine position with both lower limbs flexed naturally. Keeping the upper body fixed, both lower limbs were rotated to one side to the maximum curvature, held for 5 seconds, and then returned to the neutral position. This process is repeated on the other side. The entire process was repeated 10 times.
5. In the prone position, patients slowly propped up their upper body with both arms to the maximum posterior extension curvature of the lumbar spine and held this position for 30 seconds.

The exercises described above constitute a single set. These exercises were performed thrice a week, with each course lasting 2 weeks, for 3 courses over 6 weeks.

Participants in the control group were administered celecoxib capsules (0.2 g, Jiangsu Zhengda) as oral analgesic medication when necessary. Each patient received one box at enrollment and the reasons for and duration of use were recorded. Medication use was necessary if pain NRS scores exceeded 7 for more than 24 hours without relief or if pain prevented or discouraged exercise.

### 2.3. Outcomes

#### 2.3.1. Primary outcome

The NRS was used to evaluate patients’ pain before treatment; at weeks 2, 4, and 6 of the treatment period; and at week 12 of the follow-up period.

#### 2.3.2. Secondary and supporting outcomes

**Figure 3.**
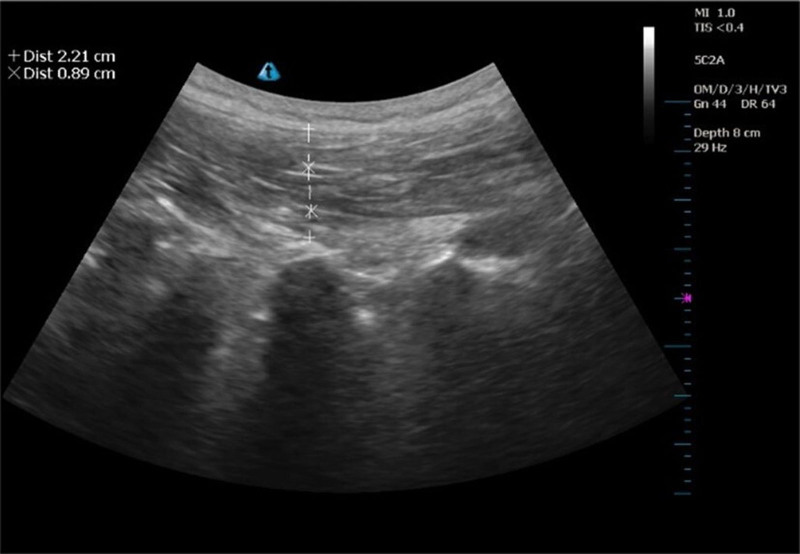
Measurement of the multifidus muscle.

1. ODI, Japanese Orthopedic Association (JOA) scores were used to evaluate the patients’ dysfunction and the lumbar spine function before treatment; at weeks 2, 4, and 6 during treatment; and at week 12 during follow-up.
2. MU assessment of the 4th lumbar multifidus muscle thickness was performed at baseline, week 6, and week 12. The multifidus muscle thickness was measured in degrees, the maximum value was recorded, and the measurements were repeated 3 times for averaging (Fig. 3).

### 2.4. Safety assessments

The safety metric for this clinical trial was the incidence of adverse events. Adverse events that may occur during the course of this study included subcutaneous bleeding, bruising, hematoma after needle-knife release, localized skin redness, swelling, and itching that may occur after postoperative infection of the wound with water.

### 2.5. Sample size calculation

A review of the literature^[22,25]^ and preliminary findings indicate that the mean NRS score of the control group at the conclusion of the final treatment was 4.70 ± 3.10 points. It was anticipated that the NRS score of the observation group could be reduced by 2.60 points. The sample size estimation was conducted using the Power Analysis and Sample Size 2021 software, with a two-sided α of 0.05, 1-β of 90%, resulting in a sample size of n = 31 cases. To account for a 5% loss and refusal rate, a total of 33 study subjects were required in each group, yielding a total of 66 cases.

### 2.6. Statistical analysis

#### 2.6.1. Trial analysis data set

1. Full Analysis Set (FAS): The FAS population is defined as all patients who have been randomized, entered treatment, received at least one treatment, and have relevant efficacy evaluations. In the event of missing data in the efficacy-related portion of the FAS set, the last previous observation carry-over (LOCF) method will be employed for data supplementation.
2. Per-Protocol Set (PPS): The PP population is defined as cases that have completed the specified treatment regimen in accordance with the protocol, have not exhibited any significant protocol deviations, and have completed all elements of the evaluation. The dataset for these cases will be determined at the time of data review after completion of the trial.

#### 2.6.2. Statistical data processing

1. The per-protocol analysis was ultimately selected for this trial’s data analysis. Normally distributed quantitative data were presented as mean ± standard deviation (± SD), and non-normally distributed data were presented as median (interquartile range) and M (IQR). Qualitative data are presented as frequencies (composition ratios) (n (%)).
2. Qualitative dichotomous data were analyzed using χ^2^ test. The normality of the quantitative data was evaluated, and when the data were not normally distributed, equivalent nonparametric tests were employed. Effect sizes were calculated for between-group comparisons to help determine the reliability of the results. Quantitative data were subjected to repeated-measures analysis of variance (ANOVA) only if they satisfied the chi-square and normal distribution criteria. and calculating effect sizes to judge the reliability of the results.
3. All the data of this study were processed by SPSS statistic 25.0 software for statistical analysis to produce the results. All statistical tests were performed using two-sided test and the significance level was taken as α = 0.05, with *P* < .05 being considered statistically significant. We interpreted Cohen’s *d* values with the classification proposed where an effect size of 0.2 means a small effect, 0.5 means a medium effect, 0.8 means a large effect.

## 3. Results

### 3.1. Trial enrollment and completion

From March 2023 to October 2023, 66 eligible patients were included based on strict adherence to the inclusion criteria. Of these, 62 patients completed the entire treatment and follow-up periods, comprising 32 and 30 patients in the observation and control groups, respectively.

### 3.2. Comparison of general information of participants at baseline

In this study, no significant differences were observed between the groups in terms of sex, age, body mass index, LBP duration, NRS, ODI, JOA, and the 4th lumbar multifidus muscle thickness (*P* > .05). The baseline information is presented in Table 1.

**Table 1:** Basic information of patients

Groups	Observation (n = 33)	Control (n = 33)	χ² value/*t* value/*Z* value	*P* value
Gender (m/f) (n)	15/18	16/17	0.061 ^a1^	.805^a2^
Age (years) x ¯±SD	46.97 ± 11.48	46.06 ± 7.97	0.374	.710
BMI (kg/m^2^) $\left(\bar{x}\pm SD\right)$	24.40 ± 2.62	24.59 ± 3.18	-0.275	.784
Duration of low back pain (years) (M, (IQR))	5.00 (3.50,7.00)	5.00 (4.00, 6.00)	-0.045 ^b1^	.964^b2^
NRS (M, (IQR))	6.00 (5.00,7.00)	6.00 (5.00,7.00)	-0.446^c1^	.656 ^c2^
ODI $\left(\bar{x}\pm SD\right)$	21.92 ± 3.827	21.82 ± 3.311	0.103	.918
JOA $\left(\bar{x}\pm SD\right)$	18.61 ± 2.397	18.48 ± 2.033	0.222	.825
L4MF thickness (cm) $\left(\bar{x}\pm SD\right)$	2.27 ± 0.365	2.19 ± 0.341	0.815	.418

a1: χ² values, a2: using chi-square test.

b1, c1: *Z* values, b2, c2: using Wilcoxon Mann–Whitney test.

The other 2 independent sample *t* test was used and the data are *t* values, with *P* > .05 representing no statistically significant difference.

$\bar{x}$ = mean, M = median, IQR = interquartile range, JOA = Japanese Orthopedic Association, L4MF = 4th lumbar vertebrae multifidus muscle, NRS = Numerical Rating Scale, ODI = Oswestry Disability Index, SD = standard deviation.

### 3.3. Primary outcome

The pretreatment median NRS score of patients in the 2 groups was 6.00 (IQR: 5.00, 7.00) (Tables 1 and 2), and the difference was not statistically significant according to the U test (*Z* = −0.446, *P* = .656), indicating comparability between the groups (Table 2). Two weeks after treatment, the difference in NRS scores was not statistically significant (*Z*_2weeks post-treatment_ = −1.436, *P* > .05). The differences in NRS scores at 4, 6, and 12 weeks after treatment were statistically significant (*Z*_4 weeks post-treatment_ = −3.522, *Z*_6 weeks post-treatment_ = −4.069, *Z*_6 weeks post-treatment_ = −4.965, *P* < .05) (Table 2). The median NRS scores in the observation group were lower than those in the control group, indicating more significant pain improvement and efficacy in the observation group.

**Table 2:** Comparison of NRS scores during treatment

Groups	Pretreatment	1st treatment (week 2)	2nd treatment (week 4)	Post-final treatment (week 6)	Follow-up period (week 12)	χ^2^
Observation	6.00 (5.00, 7.00)	4.00 (3.25, 5.00)	3.00 (3.00, 3.75)	2.00 (1.00, 2.00)	2.00 (1.00, 2.00)	119.200†
Control	6.00 (5.00, 7.00)	5.00 (4.00, 5.00)	4.00 (3.00, 5.00)	3.00 (2.00, 4.00)	3.00 (2.00, 4.00)	94.805†
*Z*	−0.446	−1.436	−3.522*	−4.069*	−4.965*	/

NRS = Numerical Rating Scale.

* *P* < .05 compared with the control group.

† *P* < .05 for the overall comparison within the group.

**Figure 4.**
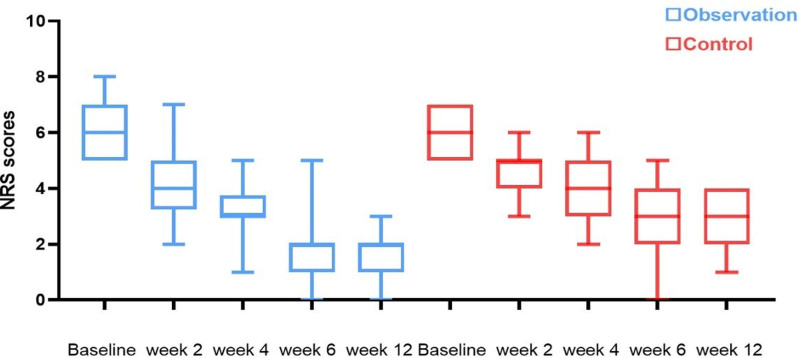
Trend graph of NRS score changes during treatment in both groups. NRS = Numerical Rating Scale.

Friedman test analysis showed that the median NRS scores of patients in both groups gradually decreased with time, with statistically significant differences in each group (χ²_Observation_ = 119.200, χ²_Control_ = 94.805, *P* < .05) (Table 2). NRS scores decreased in both groups at 2, 4, 6, and 12 weeks after treatment. From the 4th week onward, the differences in NRS scores were statistically significant, with the observation group showing greater improvement in pain than the control group (Table 2; Figure 4).

The Wilcoxon signed-rank test showed that the NRS pain scores in both groups progressively decreased with increasing treatment time. In the observation group, the NRS scores gradually decreased from the second treatment to 12 weeks post-treatment (*Z*_a1_ = −4.888, *Z*_a2_ = −4.988, *Z*_a3_ = −4.988, *Z*_a4_ = −4.989, *P* < .05) (Table 3). However, the difference in NRS scores after the last treatment during the follow-up period was not statistically significant (*Z*_a5_ = −1.134, *P* = .257 > 0.05) (Table 3). In the control group, NRS scores gradually decreased with increasing treatment time from the second treatment to 12 weeks post-treatment (*Z*_b1_ = −4.736, *Z*_b2_ = −4.792, *Z*_b3_ = −4.744, *Z*_b4_ = −4.834, *P* < .05). After stopping treatment, the difference in NRS scores compared to the last treatment was not statistically significant during the follow-up period (*Z*_b5_ = −0.275, *P* = .783 > 0.05) (Table 3).

**Table 3:** Comparison of NRS scores at different time points

Time point in comparison	Test results	Observation	Control
Post-treatment week 2 vs baseline	Negative rank mean	15.50	14.50
	*Z*	−4.888*_a1_	−4.736*_b1_
Post-treatment week 4 vs baseline	Negative rank mean	16.50	15.00
	*Z*	−4.988*_a2_	−4.792*_b2_
Post-treatment week 6 vs baseline	Negative rank mean	16.50	15.00
	*Z*	−4.988*_a3_	−4.744*_b3_
Week 12 post-treatment vs baseline	Negative rank mean	16.50	15.50
	*Z*	−4.989*_a4_	−4.834*_b4_
Final treatment and follow-up	Negative rank mean	2.67 (2.00 ^c1^)	11.06 (9.23 ^c2^)
	*Z*	−1.134 _a5_	−0.275 _b5_

Negative rank means were recorded because positive ranks were mostly 0. c1 and c2 are positive rank means.

NRS = Numerical Rating Scale.

* Within-group comparisons, *P* < .05.

### 3.4. Secondary and supporting outcomes

#### 3.4.1. Comparison of ODI dysfunction scores between the 2 groups before and after treatment and during follow-up

**Figure 5.**
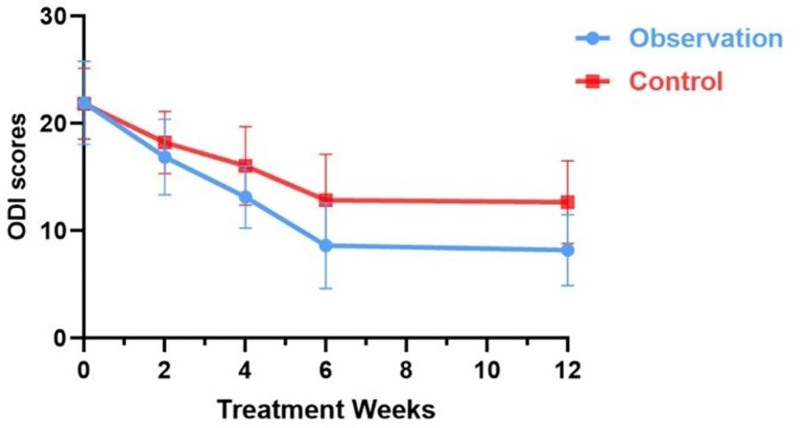
Trend graph of ODI score changes during treatment in both groups. ODI = Oswestry Disability Index.

After removing the data, the pretreatment ODI dysfunction scores of the 2 groups of patients were 21.94 ± 3.885 and 21.87 ± 3.309, respectively. The difference was not statistically significant according to the ANOVA (*t* = 0.077, *P* > .05), indicating that the groups were comparable (Table 4). The difference in ODI dysfunction scores at 2 weeks after treatment was not statistically significant (*t*_2 weeks post-treatment_ = −1.648, *P* > .05, *d* = 0.42); however, the difference at 4, 6, and 12 weeks after treatment was statistically significant (*t*_4 weeks post-treatment_ = −3.474, *t*_6 weeks post-treatment_ = −4.034, and *t*_12 weeks post-treatment_ = −4.923, *P* < .05, *d* > 0.8) (Table 4). The ODI scores of the observation group were lower, indicating a significant improvement in patient dysfunction (Fig. 5). Cohen’s *d*-value > 0.8 proves the high reliability of the results.

**Table 4:** Comparison of ODI scores during treatment

Groups	Pretreatment	1st treatment (2 weeks)	2nd treatment (4 weeks)	Post-final treatment (6 weeks)	Follow-up period (12 weeks)	*F*
Observation	21.94 ± 3.885	16.88 ± 3.526	13.16 ± 2.908	8.63 ± 3.998	8.22 ± 3.299	99.428†
Control	21.87 ± 3.309	18.23 ± 2.909	16.07 ± 3.667	12.87 ± 4.281	12.70 ± 3.861	88.591†
*t*	0.077	−1.648	−3.474*	−4.034*	−4.923*	/

ODI = Oswestry Disability Index.

* *P* < .05 compared with the control group.

† *P* < .05 for overall comparison within the group.

ODI scores in both groups decreased at 2, 4, 6, and 12 weeks after treatment, with statistically significant differences in the one-way repeated-measures ANOVA test (*F*_Observation_ = 99.428, *F*_Control_ = 88.591, *P* < .05) (Table 4). Both groups showed a gradual decline in ODI scores over time, with the observation group demonstrating better functional improvement than the control group (Fig. 5).

Compared with the pretreatment period, the observation group showed a gradual decrease in ODI scores from the second treatment to 12 weeks after treatment (*t*_a1_ = 9.853, *t*_a2_ = 12.425, *t*_a3_ = 16.285, *t*_a4_ = 18.350, *P* < .05). The differences in the ODI scores with the final treatment were not statistically significant after treatment discontinuation during the follow-up (*t*_a5_ = 1.200, *P* > .05) (Table 5) (Table 5). In the control group, from the second treatment to 12 weeks after treatment, the ODI scores gradually decreased with prolonged treatment time (*t*_b1_ = 9.664, *t*_b2_ = 10.890, *t*_b3_ = 10.856, *t*_b4_ = 14.703, *P* < .05), and there was no statistically significant difference between the ODI scores at follow-up and at the end of treatment (*t*_b5_ = 0.163, *P* > .05) (Table 5).

**Table 5:** Comparison of ODI scores at different time points

Time point in comparison	Test results	Observation	Control
Post-treatment week 2 vs baseline	Difference (the result of subtraction)	5.063 ± 2.906	3.633 ± 2.059
	*t*	9.853*_a1_	9.664*_b1_
Post-treatment week 4 vs baseline	Difference (the result of subtraction)	8.781 ± 3.998	5.800 ± 2.917
	*t*	12.425*_a2_	10.890*_b2_
Post-treatment week 6 vs baseline	Difference (the result of subtraction)	13.313 ± 4.624	9.000 ± 4.541
	*t*	16.285*_a3_	10.856*_b3_
Post-treatment week 12 vs baseline	Difference (the result of subtraction)	13.719 ± 4.229	9.167 ± 3.415
	*t*	18.350*_a4_	14.703*_b4_
Final treatment and follow-up	Difference (the result of subtraction)	0.406 ± 1.915	0.167 ± 5.615
	*t*	1.200 _a5_	0.163 _b5_

ODI = Oswestry Disability Index.

* Within-group comparisons, *P* < .05.

#### 3.4.2. Comparison of JOA efficacy assessment scores between the 2 groups before and after treatment and during follow-up

**Figure 6.**
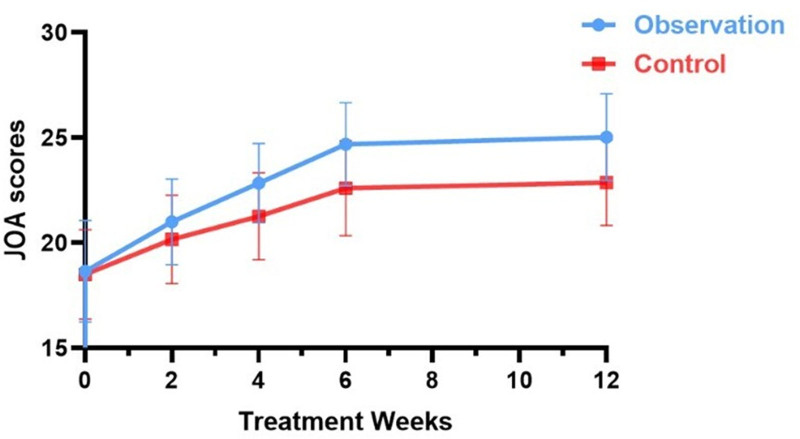
Trend graph of JOA dysfunction scores during the treatment period in both groups. JOA = Japanese Orthopedic Association.

After removing the data, the pretreatment JOA scores of patients in the 2 groups were 18.66 ± 2.418 and 18.50 ± 2.129, respectively, and the differences were not statistically significant by ANOVA (*t* = 0.269, *P* > .05), indicating comparability between the groups (Table 6). The difference in JOA scores at 2 weeks after treatment was not statistically significant (*t*_2 weeks post-treatment_ = 1.581, *P* > .05, *d* = 0.40); however, the difference in JOA scores was statistically significant when comparing the 2 groups at 4, 6, and 12 weeks after treatment (*t*_4 weeks post-treatment_ = 3.142, *t*_6 weeks post-treatment_ = 3.872, and *t*_12 weeks post-treatment_ = 4.309, *P* < .05, *d* > 0.8) (Table 6), with higher JOA scores in the observation group, indicating better improvement in patient function (Fig. 6). Cohen’s *d* value > 0.8 proves the high reliability of the results.

**Table 6:** Comparison of JOA scores during treatment

Groups	pretreatment	1st treatment (2 weeks)	2nd treatment (4 weeks)	Post-final treatment (6 weeks)	follow-up period (12 weeks)	*F*
Observation	18.66 ± 2.418	21.00 ± 2.048	22.84 ± 1.886	24.69 ± 1.975	25.03 ± 2.063	107.705†
Control	18.50 ± 2.129	20.17 ± 2.102	21.27 ± 2.067	22.60 ± 2.268	22.87 ± 2.036	33.614†
*t*	0.269	1.581	3.142*	3.872*	4.309*	/

JOA = Japanese Orthopedic Association.

* *P* < .05 compared with the control group.

† *P* < .05 for overall comparison within the group.

JOA scores gradually increased in both groups as treatment progressed. Compared with pretreatment, JOA scores increased in both groups at 2, 4, 6, and 12 weeks after treatment, with a statistically significant difference in the one-way repeated-measures ANOVA test (*F*_Observation_ = 107.705, *F*_Control_ = 33.614, *P* < .05) (Table 6). The observation group showed better improvement in functional status than the control group (Fig. 6).

Compared to pretreatment, JOA scores in the observation group increased progressively with increasing treatment time from the second treatment to 12 weeks post-treatment (*t*_a1_ = −8.873, *t*_a2_ = −13.132, *t*_a3_ = −19.095, *t*_a4_ = −17.123, *P* < .05), and the difference in JOA scores from the last treatment remained statistically significant (*t*_a5_ = −2.075, *P* < .05) (Table 7). In the control group, JOA scores gradually increased from the second treatment to 12 weeks after treatment (*t*_b1_ = −6.210, *t*_b2_ = −8.537, *t*_b3_ = −8.104, *t*_b4_ = −11.909, *P* < .05), and the difference between the JOA scores and the final treatment was not statistically significant after stopping the treatment during follow-up (*t*_b5_ = −0.603, *P* > .05) (Table 7).

**Table 7:** Comparison of JOA scores at different time points

Time point in comparison	Test results	Observation	Control
Post-treatment week 2 vs baseline	Difference (the result of subtraction)	−2.344 ± 1.494	−1.667 ± 1.470
	*t*	−8.873*_a1_	−6.210*_b1_
Post-treatment week 4 vs baseline	Difference (the result of subtraction)	−4.188 ± 1.804	−2.767 ± 1.775
	*t*	−13.132*_a2_	−8.537*_b2_
Post-treatment week 6 vs baseline	Difference (the result of subtraction)	−6.031 ± 1.787	−4.100 ± 2.771
	*t*	−19.095*_a3_	−8.104*_b3_
Week 12 post-treatment vs baseline	Difference (the result of subtraction)	−6.375 ± 2.106	−0.267 ± 2.420
	*t*	−17.123*_a4_	−11.909*_b4_
Final treatment and follow-up	Difference (the result of subtraction)	−0.344 ± 0.937	−4.367 ± 2.008
	*t*	−2.075*_a5_	−0.603 _b5_

JOA = Japanese Orthopedic Association.

* *P* < .05.

#### 3.4.3. Comparison of L4 Multifidus Muscle (L4MF) thickness before and after treatment and during follow-up period between 2 groups of patients

**Figure 7.**
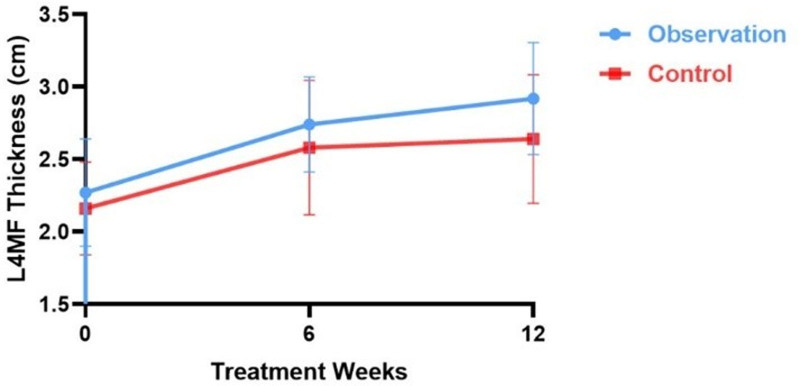
Trend of multifidus muscle thickness change before and after treatment in both groups.

After removing the data, the pretreatment thickness of the L4 multifidus muscle in both groups of patients was 2.27 ± 0.370 and 2.16 ± 0.321, respectively, and the difference was not statistically significant after ANOVA (*t* = 1.97, *P* > .05), indicating comparability between the groups (Table 8). As treatment progressed, the thickness of the L4 multifidus muscle increased in both the groups (Table 8; Fig. 7). At 6 weeks post-treatment, the difference in thickness was not statistically significant (*t*_6 weeks post-treatment_ = 1.650, *P* > .05, *d* = 0.40). However, at follow-up, the difference was statistically significant (*t*_6 weeks post-treatment_ = 2.536, *P* < .05, *d* = 0.64), and the observation group showed a greater increase in muscle thickness than the control group, indicating more pronounced efficacy in the observation group (Table 8; Fig. 7). Cohen’s *d* value = 0.64 attests to the reliability of the results with a medium effect.

**Table 8:** Comparison of L4 multifidus muscle thickness during treatment

Groups	Before treatment (cm)	After last treatment (cm)	Follow-up period (cm)	*F*
Observation	2.27 ± 0.370	2.74 ± 0.329	2.92 ± 0.387	19.059†
Control	2.16 ± 0.321	2.58 ± 0.465	2.64 ± 0.444	66.625†
*t*	1.197	1.650	2.536*	/

* *P* < .05 compared with the control group.

† *P *< .05 for overall comparison within the group.

L4 MF thickness gradually increased as treatment progressed in both the groups. Compared with pretreatment, multifidus muscle thickness increased in both groups at 6 and 12 weeks after treatment, with a statistically significant difference in the one-way repeated measures ANOVA test (*F*_Observation_ =19.059, *F*_Control_ =66.625, *P* < .05) (Table 8). The observation group showed greater improvement than did the control group (Fig. 7).

Compared to the pretreatment period, the L4MF thickness gradually increased as treatment progressed from 6 to 12 weeks after treatment in the observation group (*t*_a1_ = −0.790, *t*_a2_ = −11.726, *P* < .05). And the difference in L4MF thickness after the last treatment was statistically significant after stopping treatment during the follow-up period (*t*_a3_ = −5.181, *P* < .05) (Table 9; Fig. 7), indicating further increasing in the thickness of L4MF. In the control group, from 6 to 12 weeks after treatment, L4MF thickness increased gradually as treatment progressed (*t*_b1_ = −4.495, *t*_b2_ = −6.269, *P* < .05) (Table 9; Fig. 7). and the difference in L4MF thickness after the last treatment was not statistically significant after stopping treatment during the follow-up period (*t*_b3_ = −1.113, *P* > .05) (Table 9).

**Table 9:** Comparison of L4 multifidus muscle thickness at different time points

Time point in comparison	Test results	Observation	Control
Post-treatment week 6 vs baseline	Difference (the result of subtraction)	−0.480 ± 0.277	−0.418 ± 0.509
	*t*	−9.790* a1	−4.495* b1
Week 12 post-treatment vs baseline	Difference (the result of subtraction)	−0.648 ± 0.313	−0.486 ± 0.424
	*t*	−11.726* a3	−6.269* b2
Final treatment and follow-up	Difference (the result of subtraction)	−0.168 ± 0.183	−0.068 ± 0.333
	*t*	−5.181* a3	−1.113 b3

* *P* < .05.

### 3.5. Safety comparison

One subject in the observation group was unable to continue to participate in follow-up treatment due to a change in job, and 1 case was dislodged. In the control group, 2 subjects gave up the follow-up treatment because of the lack of significant pain relief, and 1 subject missed the visit, and 2 cases were discharged. The patients in the observation group experienced soreness and pain in the wounds after the needle knife loosening treatment, which improved after 3 times of localized hot compresses and did not affect the follow-up treatment; no adverse reactions were observed in the subjects in the control group. There was no significant difference in the incidence of adverse reactions between the 2 groups (*P* > .05).

## 4. Discussion

The study included patients aged 32 to 66 years with a mean age of 46 years. The percentage of female patients was 53.03%, and the disease duration in both groups ranged from 2 to 10 years, with a median of 5 years in both groups (observer group: 5 [3.5, 7]; control group: 5 [4, 6]), which is consistent with the current epidemiological findings of CNSLBP.

Pain and lumbar spine dysfunction are the key features of RNSLBP, with no difference in pain levels between day and night, no significant relief from bed rest, and pain scores between 5 and 8 (moderate to severe pain), mostly affecting night sleep. This dysfunction, characterized by the inability to sit, walk, and stand for long periods, severely limits the patient’s daily life and work.

NRS is a commonly used, easy-to-operate, and intuitive pain assessment scale in clinical practice with high accuracy and is rarely affected by non-pain intensity factors.^[26]^ After treating the 2 groups of patients, the NRS scores gradually decreased over time (*P* < .05). After the final treatment and follow-up period, the NRS scores showed statistically significant differences (*P* < .05). The time effect difference between the groups was also significant (*P* < .05), indicating that the observation group had a more substantial decline in the NRS scores over time. The MU-guided needle-knife loosening treatment effectively relieved pain in patients with RNSLBP.

The ODI is a research scale used to assess the functional status of patients with LBP based on subjective evaluation of CLBP symptoms and functions. After treatment, the ODI scores of the 2 groups of patients gradually decreased as treatment continued (*P* < .05), with statistically significant differences between the 2 groups at the end of the treatment (*P* < .05). The decrease in ODI scores in the observation group was better than that in the control group, suggesting that needle-knife treatment of the multifidus muscle improved disability in patients with RNSLBP.

The JOA is an effective evaluation scale for assessing improvements in lumbar spinal function. After treating the 2 groups of patients, the JOA score gradually increased as treatment progressed (*P* < .05), with a statistically significant difference between the 2 groups at the end of treatment (*P* < .05). After the treatment ended, patients in the control group experienced different degrees of lumbar pain recurrence, whereas patients in the observation group experienced long-term treatment efficacy. Therefore, MU-guided needle-knife loosening treatment for patients with RNSLBP has better clinical efficacy than the usual care.

The difference in L4MF thickness was not statistically significant after the last treatment (*P* > .05); however, 12 weeks after treatment, the difference was statistically significant (*P* < .05), indicating that the recovery of multifidus muscle function in patients with RNSLBP takes longer period. However, needle knife loosening activates the multifidus muscle and improves its morphology and function. There was a time and group interaction in multifidus muscle thickness, with similar increases in both groups. However, the difference in thickness between the groups was statistically significant at follow-up (*P* < .05), suggesting that a longer follow-up may be needed to observe changes. The current analysis shows that MU-guided needle-knife loosening treatment for L4MF has a positive therapeutic effect on multifidus muscle function.

NRS, ODI, JOA, and L4MF thickness indicated that the observation group had better clinical efficacy and that MU-guided needle-knife loosening therapy was more effective than usual care in relieving pain symptoms in patients with RNSLBP.

Although the anatomical cause of RNSLBP is unclear, diminished or insufficient motor control of the deep lumbar spine muscles has been recognized as a key etiological factor.^[27]^ Various imaging studies have been performed to observe multifidus muscle morphology in patients with CLBP. Kamaz et al.^[28]^ used CT to observe the morphology of the paraspinal muscles in patients with CLBP and identified significant muscle atrophy, which was significantly greater than that in the other paraspinal muscles. Fuwei et al^[29]^ confirmed the presence of fatty infiltration and atrophy of multifidus muscle in patients with CNSLBP using MR imaging scanning technique. Additionally, clinical studies involving MR imaging have highlighted the highest levels of fatty infiltration and atrophy in the L4-5 segment of the multifidus muscles.^[30]^ The multifidus muscle of patients with RNSLBP usually has strong linear or punctate strong echoes, inhomogeneous low-signal areas, and intramuscular interstitial spaces that are faintly feathery, gridded, or do not appear on MU imaging. These findings suggest muscle atrophy, fatty infiltration, and scarring of muscle fibers.^[31]^ NSLBP patients usually experience altered patterns of lumbar multifidus muscle recuperation, endurance deficits, decreased repositioning accuracy, and delayed transversus abdominis muscle activation.^[27]^ Patients with CNSLBP commonly have fat infiltration and muscle atrophy in the low back muscle group, and the results of the study indicated that the reduction of clinical symptoms in patients was accompanied by an increase in the thickness of the multifidus muscle, and the follow-up data were statistically significant, so we can speculate that increasing the thickness of the multifidus muscle can improve the clinical symptoms of the patients. Consequently, new treatments now focus on improving muscle function, particularly the multifidus muscles.

Mechanical stimulation of the multifidus muscle using a needle knife stimulates the local ischemic multifidus muscle by loosening and peeling off the adhesion and contracture of the lesion area, promoting the regeneration of capillaries, restoring part of the cellular blood flow, enhancing local microcirculation, reducing fatty infiltration of the multifidus muscle, and improving the mechanical balance of the lumbar spine.^[32,33]^ The needle knife also promotes benign proliferation of skeletal muscle,^[34]^ accelerates fibrinolysis and inflammation absorption,^[35]^ and effectively controls inflammation and reduces pain.

In this trial, due to the unavailability of interventions that could emulate the needle knife, a single-blind design was employed, which may introduce bias, particularly in subjective outcome indicators such as NRS, ODI, and JOA. Patients’ knowledge of the intervention modality may have brought about a more positive subjective disposition toward the intervention in the observation group. The unblinding of the performers may lead to the performers unconsciously taking a tendency color and generating performance bias. To mitigate this, the trial performers were separated from the evaluators, ensuring minimal information bias and accurate evaluation. Similarly, the evaluators were separated from the data analysts, guaranteeing objective statistical results. Furthermore, an objective indicator, multifidus muscle thickness, was incorporated to assist in the assessment of the treatment effect and to maximize the reliability of the results.

The per-protocol analysis was ultimately selected for this trial’s data analysis. Of the missing data, the patients in the observation group failed to complete the post-treatment efficacy evaluation following one treatment session and could not be included in the FAS. Of the 3 remaining control patients, the patients demonstrated poor adherence, used non-trial-provided pain medication due to unresolved pain, and it was not possible to ascertain whether they proceeded with the continuation of the treatment; as a result, they were excluded. Ultimately, due to the limited amount of missing data, primarily within the control group of usual care based on prior outcomes, the patients in the control group underwent conventional conservative treatment with unsatisfactory results. Non-adherent patients may adopt more effective treatments for pain relief than usual care, which, if not excluded, may increase the efficacy of usual care, thereby increasing the effectiveness of usual care and decreasing the significance of between-group comparisons, affecting the results of the experiment. This was done to prevent the introduction of bias into the data through the filling in of missing values. Consequently, the PP analysis was selected. However, since PP analysis excluded patients with poor adherence, which could lead to an exaggeration of the true efficacy, and since PPS is a subset of FAS, the incompleteness of the statistical analysis could also affect the reliability of the results.

This study had certain limitations, including a short clinical observation period and a small sample size. Further multicenter, large-sample, long-term studies, multivariate statistical analysis methods are needed to verify the clinical efficacy of this treatment.

## 5. Conclusion

This study showed that the NRS, ODI, and JOA scores improved more with MU-guided needle knife loosening treatment for RNSLBP than with the usual care. The needle knife effectively enhanced the lumbar spine function and reduced pain. Additionally, MU-guided needle-knife treatment is efficient and safe, has a shorter clinical treatment period and fewer side effects, making it suitable for broader clinical use.

## Acknowledgments

We would like to thank Editage (www.editage.cn) for English language editing.

## Author contributions

**Conceptualization:** Xia Li, Zhanying Tang.

**Data curation:** Sidi Zhang.

**Formal analysis:** Mingqi Wu.

**Investigation:** Xia Li.

**Methodology:** Xia Li, Hongkai Zhang, Zhanying Tang.

**Project administration:** Xia Li.

**Software:** Sidi Zhang, Mingqi Wu.

**Supervision:** Shiyun Wang, Zhanying Tang, Jing Xiao.

**Writing – original draft:** Xia Li, Hongkai Zhang.

**Writing – review & editing:** Zhanying Tang, Jing Xiao.
